# Building Interest in Cardiothoracic Surgery at an Osteopathic Medical School: Results of an Institutional Study and a Guide for Medical Schools

**DOI:** 10.7759/cureus.49471

**Published:** 2023-11-27

**Authors:** Andrew D Vogel, Austin Wynn, Michelle Sindoni, Megan C Richards, Adam R Eppler, Caleb L Hamilton, Juan J Gallegos, Tyler J Wallen

**Affiliations:** 1 Department of Research, Alabama College of Osteopathic Medicine, Dothan, USA; 2 Department of Cardiothoracic Surgery, Southeast Health Medical Center, Dothan, USA; 3 Department of Cardiothoracic Surgery, Huntsville Hospital Health System, Huntsville, USA; 4 Department of Cardiothoracic Surgery, Geisinger Commonwealth School of Medicine, Wilkes-Barre, USA

**Keywords:** simulation, osteopathic medicine, interest groups, medical students, cardiothoracic surgery, surgical education

## Abstract

Objective

A previous study at this institution revealed a connection between interest group involvement and specialty interest while identifying the negative perceptions of cardiothoracic (CT) surgery. This study aimed to build interest and ameliorate the negative perceptions of CT surgery by exposing pre-clinical students to the field through engaging events.

Methods

Students at a US osteopathic institution who attended CT surgery committee events were invited to complete an online survey after each event. Associations between the number of events attended and ranked responses to survey questions were assessed by two-tailed Spearman correlations. Statistical comparisons in ranked responses between the events attended and the survey questions were assessed by a two-way analysis of variance (ANOVA). Pre-clinical students actively enrolled at the institution during the 2022-2023 academic year were eligible for inclusion.

Results

There were 83 surveys completed over seven events. There was a significant association between the number of events a student attended and their perception of CT surgeon's work/life balance with a correlation coefficient of .258 (P=0.019) and whether CT surgeons have time for their families with a correlation coefficient of .235 (P=0.035). Residents and medical student events as well as wet lab events increased interest the most and helped students feel equipped to apply for CT surgery.

Conclusions

While negative perceptions associated with CT surgery exist, these may be ameliorated with more exposure to the field. Unique events that expose pre-clinical students to multiple facets of CT surgery, including physicians and trainees in the field, as well as offering hands-on activities, may increase interest in the field and further pursuit of the field during clinical years.

## Introduction

Cardiothoracic (CT) surgery is a surgical subspecialty that struggles to attract medical students. As the population continues to age, there will be an increased need for CT surgeons. However, as interest in the field continues to decrease among medical students, it will lead to an increase in caseload on CT surgeons [1-3]. Common factors have been identified that distract medical students from the field including work/life balance, length of residency training, lack of family time, and personality of CT surgeons [4,5]. Previous studies exploring the perceptions and attitudes medical students have towards CT surgery were completed at large allopathic academic institutions until recently [3,4,6-10]. A previous institutional study by this group demonstrated similar data to that exhibited at these large institutions. Despite these results, there has been a disproportionately low number of osteopathic students applying to CT surgery residencies. This study developed the connection between interest group involvement and specialty of interest in students during their first and second years of medical school and suggested the need for the further engagement of medical students early in their careers [5].

This study is the second in a series that has assessed osteopathic medical student's interest in CT surgery from one institution at the beginning of the academic year, will determine the impact of a CT surgery committee on pre-clinical student's interest in the field, and will finally nationally assess osteopathic medical student's interest in CT surgery who are members of the American College of Osteopathic Surgeons-Medical Student Section (ACOS-MSS). The aim of this study was to expose students, build interest, and ameliorate the negative perceptions associated with CT surgery. More importantly, by exposing students to CT surgery through engaging and innovative events, we hope to develop a model for other medical schools not connected to large university-affiliated institutions who wish to start a CT surgery interest group of their own.

## Materials and methods

The Institutional Review Board of the Alabama College of Osteopathic Medicine classified this project as exempt (HS220801-E) and initially approved it on August 2, 2022. Students who attended each event took the anonymous survey voluntarily and gave written consent.

Study recruitment and design

Pre-clinical students actively enrolled at the Alabama College of Osteopathic Medicine (Dothan, AL) during the 2022-2023 academic year were eligible for inclusion. Students are subjected to a standard medical school curriculum, with the first two years dedicated to the study of medical sciences (pre-clinical students) and the second two years dedicated to the study of clinical medicine via experiential clinical rotations (clinical students). There were 410 pre-clinical students (54%) enrolled during the study period. The institution has one general surgeon and no CT surgeon on its faculty. Pre-clinical students can join and participate in the American College of Osteopathic Surgeons-Medical Student Section (ACOS-MSS) school chapter to gain exposure to general surgery, neurosurgery, urology, plastic surgery, and CT surgery. However, a CT surgery interest committee within the ACOS-MSS school chapter had not been formed thus far to ensure adequate exposure to the field. Author ADV chartered the first CT surgery interest committee under the ACOS-MSS school chapter and served as the chair of the committee where all events were conducted. All pre-clinical students had the opportunity to attend lecture events conducted by the committee, while wet lab experiences were offered to active members of the ACOS-MSS school chapter due to limited spots.

After reviewing existing survey instruments, a de novo online survey instrument was constructed with input from past literature [4-6,9]. The survey instrument questions were developed by authors ADV, AW, MS, and MCR with input and review by the co-authors. The instrument was then tested by a focus group of medical students with feedback incorporated by the survey designers as appropriate. Baseline demographics, the perception of the specific event, and the perception of CT surgery were evaluated after each event was conducted. The survey instrument questions are provided in the *Supplemental Material* in the Appendices section. The survey was created with an online survey platform and distributed to all medical students via quick-response (QR) code or email. It was then conducted across the 2022-2023 academic year with three events occurring during the fall semester and four events occurring during the spring semester. Only completed surveys were included in the data analysis.

Event design

**Figure 1:**
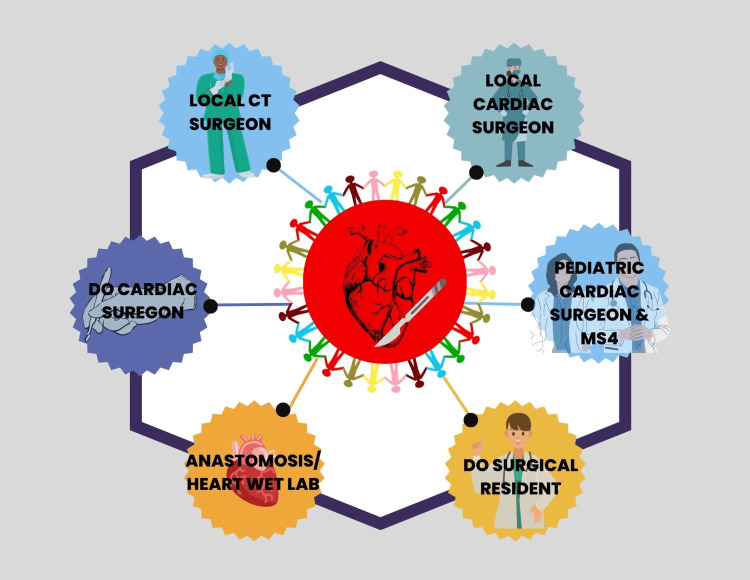
A diagram of events held by the CT surgery interest committee.

There were seven total events conducted by the CT surgery interest committee (Figure 1). First, a local CT surgeon who practices at the school's affiliated hospital but is not on faculty gave an in-person discussion on CT surgery. They explained the field of CT surgery and what their operations, clinic, and practice included. Additionally, the surgeon discussed their lifestyle, work/life balance, and the training route they took to become a CT surgeon. Second, an osteopathic cardiac surgeon (DO cardiac surgeon) gave a lecture on cardiac surgery as an osteopathic surgeon and cardiac surgery in the private practice setting. They also discussed their lifestyle and the importance of work/life balance when having a family as well as their unique path to cardiac surgery and the trials they faced as an osteopathic medical student and resident. Third, a vascular anastomosis wet lab was conducted after the ACOS-MSS chapter had completed their first suture clinic. This event taught students how to suture two vessels together using a porcine aorta model. Porcine aortas were sourced from a local slaughterhouse, and the event was conducted by the CT surgeon who spoke during the first event while ACOS-MSS chapter leadership served as assistants. Fourth, a general surgery resident and recent graduate of the institution (DO surgical resident) with an interest in thoracic surgery discussed the traditional pathway to thoracic surgery. They also focused on discussing general surgery with the audience and the integral steps that are necessary for osteopathic medical students to take to match into surgery. Fifth, a congenital cardiac surgeon and a fourth-year medical student (MS4) discussed working with pediatric patients, the subspecialty of congenital heart surgery, and current research being conducted in the field by the surgeon's lab. The MS4, who matched into an integrated CT surgery residency, discussed how students can get involved in research during their pre-clinical years and how to be successful in surgery and pediatric rotations. Sixth, a local cardiac surgeon who serves as a preceptor for the institution's third- and fourth-year medical students at one of the institution's core rotation sites gave an in-person lecture about cardiac surgery at a large community hospital and the types of procedures and equipment they use. Additionally, they gave their perception of what a preceptor expects from students rotating on CT surgery and general surgery. Lastly, a heart wet lab focused on simulating an aortic valve replacement with a prosthetic valve was conducted. The lab used porcine heart models to allow for the simulation of an aortic valve replacement that students could attempt. Porcine hearts were sourced from a local slaughterhouse, and the event was conducted by the CT surgeon who hosted the previous wet lab while ACOS-MSS chapter leadership served as assistants. Aortic valve replacement models were developed with supplies including a valve prosthetic made from rigid foam board insulation sheathing and aluminum hobby wire (Figure 1).

Statistical analysis

Associations between the number of events attended and ranked responses to survey questions were assessed by two-tailed Spearman correlations and scatter plots generated using IBM SPSS Statistics for Windows, Version 27.0 (Released 2020; IBM Corp., Armonk, New York, United States). Statistical comparisons in ranked responses between the events attended and the survey questions were assessed by a two-way analysis of variance (ANOVA), with multiple comparisons assessed by a Tukey post-hoc test and data represented by bar graphs using GraphPad Prism Version 9.5.1 for Windows (GraphPad Software, Boston, Massachusetts, United States, www.graphpad.com). Across all seven events in each category, a mean value of 4.75 was determined to be a significant marker. If there was no event that ranked a value of 4.75 for a category, then the top two events for that category are reported. Significance is indicated by an asterisk (*) when P<0.05 or by multiple asterisks (** when P<0.01; *** when P<0.001; **** when P<0.0001).

## Results

There were 83 surveys completed over seven events. No person attended all seven events. Thirty-two students attended one event, 17 students attended three events, and three students attended six events.

Students' attitudes towards CT surgery events

**Figure 2:**
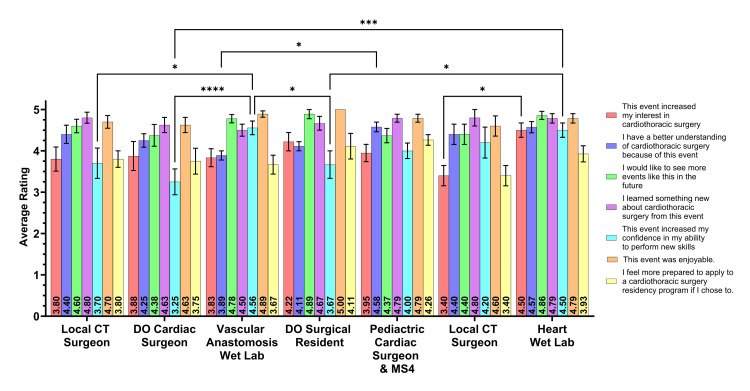
CT surgery event perceptions. CT: cardiothoracic; ANOVA: analysis of variance Student responses to questions regarding event outcomes in the post-event survey were compared between the seven different events. Each bar indicates the mean rating of participants' agreement to the corresponding-colored statements in the post-event survey, with the mean values posted in black text at the base of the bar. Error bars are representative of standard error of the mean. Significance was determined with a two-way ANOVA with multiple comparisons assessed with a Tukey post-hoc test. Significance is denoted by asterisk(s) when significant (two-tailed; P<0.05*, P<0.01**, P<0.001***, P<0.0001****).

Figure 2 displays the categories for all seven events and the Likert scale means of these categories. When looking at increased interest in CT surgery by the student, the heart wet lab was rated the highest (µ=4.50), followed by the lecture given by the DO surgical resident (µ=4.22). Students were asked if they had a better understanding of CT surgery after each event. The highest-rated events in this category were the congenital cardiac surgeon and MS4 discussion (µ=4.58) and the heart wet lab (µ=4.57). Students were then asked what events they would be interested in seeing again in the future. The highest-rated events for this question were the DO surgical resident discussion (µ=4.89), heart wet lab (µ=4.86), and vascular anastomosis wet lab (µ=4.78). The highest-rated events for learning something new about CT surgery after an event were the local CT surgeon (µ=4.80), the local cardiac surgeon (µ=4.80), the heart wet lab (µ=4.79), and the congenital cardiac surgeon and MS4 (µ=4.79). Students were then asked to rate if their confidence in the ability to perform new skills increased after each event. This category increased the most after the vascular anastomosis wet lab (µ=4.56) and the heart wet lab (µ=4.50). The most enjoyable events were the DO surgical resident discussion (µ=5.00), the vascular anastomosis wet lab (µ=4.89), and the heart wet lab (µ=4.79). Students were finally asked to rate how prepared they felt to apply to CT surgery residency programs. The highest-rated events for this category were the congenital cardiac surgeon and MS4 discussion (µ=4.26) and the DO surgical resident discussion (µ=4.11).

Significant differences were seen when comparing each category across all seven events. When looking at event perceptions (Figure 2), students felt the most confident in their skills after the vascular anastomosis wet lab when compared to the DO cardiac surgeon discussion (P<0.0001, µ=4.56 vs. µ=3.25). Likewise, students again felt more confident after the heart wet lab when compared to the DO cardiac surgeon discussion (P<0.001, µ=4.50 vs. µ=3.25). Students had a significant confidence increase after the vascular anastomosis wet lab when compared to the first local CT surgeon event (P<0.05, µ=4.56 vs. µ=3.70) and when compared to the DO surgical resident discussion (P<0.05, µ=4.56 vs. µ=3.67). Also, there was a significant difference increase in confidence of skills when comparing the heart wet lab to the DO surgical resident discussion (P<0.05, µ=4.50 vs. µ=3.67). When asked about increased interest in CT surgery, a significant difference was determined between the heart wet lab and the local cardiac surgeon event (P<0.05, µ=4.50 vs. µ=3.40). Finally, when students were asked if they had a better understanding of CT surgery, there was a significant difference between the congenital cardiac surgeon and MS4 discussion and the vascular anastomosis wet lab (P<0.05, µ=4.58 vs. µ=3.89).

Perceptions of CT surgery

**Figure 3:**
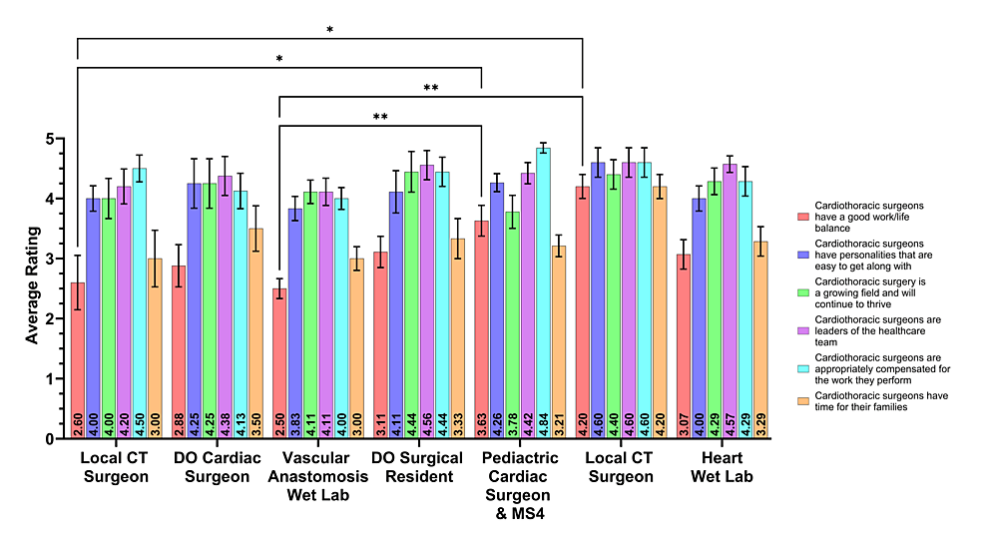
CT surgery perceptions after an event. CT: cardiothoracic; ANOVA: analysis of variance Student responses to questions in the post-event survey regarding CT surgeons were compared between the seven different events. Each bar indicates the mean rating of participants' agreement to the corresponding-colored statements in the post-event survey, with the mean values posted in black text at the base of the bar. Error bars are representative of standard error of the mean. Significance was determined with a two-way ANOVA with multiple comparisons assessed with a Tukey post-hoc test. Significance is denoted by asterisk(s) when significant (two-tailed; P<0.05*, P<0.01**, P<0.001***, P<0.0001****).

Students were then asked to rate their perceptions of CT surgeons after each event (Figure 3). When asked if CT surgeons had a good work/life balance, the highest rating was after the local cardiac surgeon discussion (µ=4.20). The highest-rated event for CT surgeons having personalities that are easy to get along with was the local cardiac surgeon event (µ=4.60). Students most likely agreed with the statement, "Cardiothoracic surgery is a growing field and will continue to thrive", after the DO surgical resident discussion (µ=4.44). Students most likely agreed that CT surgeons are leaders of the healthcare team for the local cardiac surgeon discussion (µ=4.60). Students also agreed that CT surgeons are appropriately compensated for the work they perform for the congenital cardiac surgeon and MS4 discussion (µ=4.84). Lastly, students most likely agreed that CT surgeons have time for their families for the local cardiac surgeon discussion (µ=4.20).

When looking at CT surgeon perceptions, seen in Figure 3, significant differences between events could be determined. Students rated higher for good work/life balance for the congenital cardiac surgeon and MS4 discussion compared to the vascular anastomosis wet lab (P<0.01, µ=3.63 vs. µ=2.50). Students also rated good work/life balance significantly different for the local cardiac surgeon compared to the vascular anastomosis wet lab (P<0.01, µ=4.20 vs. µ=2.50). Continuing with ratings of good work/life balance, the local cardiac surgeon was significantly higher than the local CT surgeon discussion (P<0.05, µ=4.20 vs. µ=2.60). Likewise, for the same category of good work/life balance, the congenital cardiac surgeon and MS4 discussion was significantly higher than the local CT surgeon discussion (P<0.05, µ=3.63 vs. µ=2.60). 

Event attendance and CT surgery perceptions

**Figure 4:**
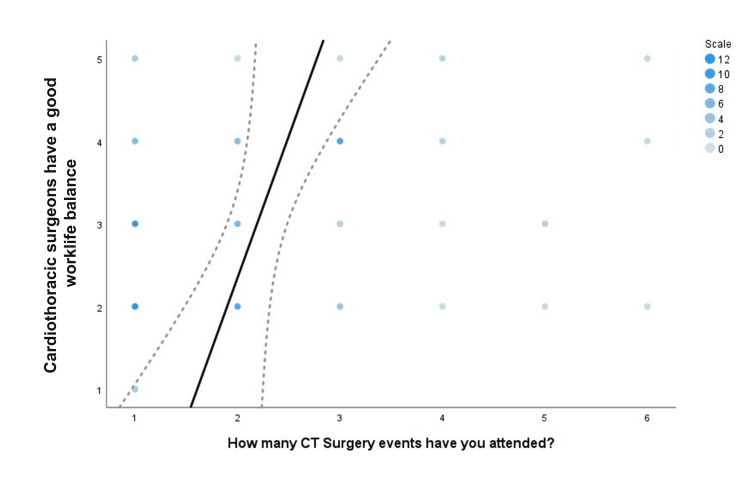
Work/life balance Spearman correlation scatter plot. The solid line illustrates the best-fit linear trend line between the number of events attended and students' ranked-response agreement with the statement, "Cardiothoracic surgeons have a good work/life balance". The intensity of blue dots is representative of the number of responses for each rank value. Dashed lines are indicative of standard error.

**Figure 5:**
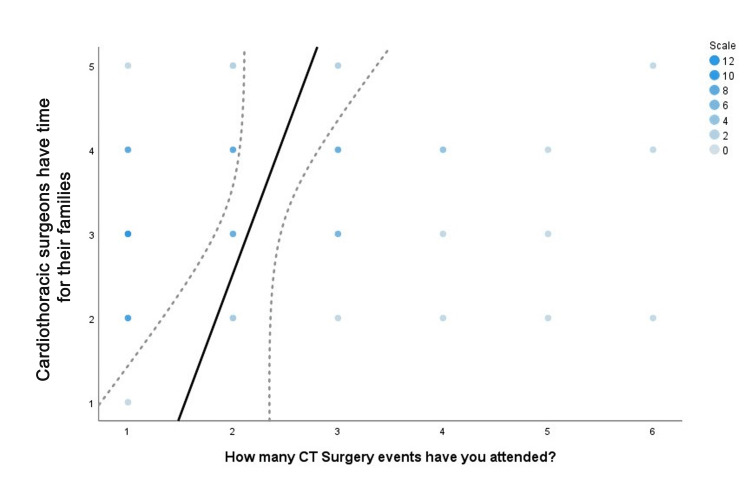
Family time Spearman correlation scatter plot. The solid line illustrates the best-fit linear trend line between the number of events attended and students' ranked-response agreement with the statement, "Cardiothoracic surgeons have time for their families". The intensity of blue dots is representative of the number of responses for each rank value. Dashed lines are indicative of standard error.

Associations between the number of events attended and students' ranked responses to perception of CT surgery were assessed. There was a significant correlation between the number of events a student attended and the perception they had of CT surgeon's work/life balance with a correlation coefficient of .258 (P=0.019) (Figure 4). Additionally, there was a significant correlation between the number of events attended and student perceptions of whether CT surgeons have time for their families with a correlation coefficient of .235 (P=0.035) (Figure 5).

## Discussion

Previous work by this group surveyed this institution and determined that negative factors associated with CT surgery include work/life balance, personality of CT surgeons, and amount of family time as a CT surgeon [5]. Many medical students have never spoken to or met a CT surgeon, and these perceptions across medical students may be rooted from rumor and stereotyping of the field. Earlier advocacy in a medical student's career may be needed to help ameliorate these perceptions [5,7,11]. Additionally, this study determined that most factors influencing a student's interest in CT surgery occurred primarily before a student entered medical school, with 83% of those interested being pre-clinical students. Furthermore, this study determined that students' specialty of interest could be influenced by the events conducted by their interest group during a student's pre-clinical years and ultimately play a role in their selection of clinical rotation electives and subsequent specialty selection [5]. With this in mind, the group sought to develop a way for students to interact with the field of CT surgery during the beginning of their medical school career and developed a CT surgery interest committee. This study aimed to determine whether interest increases in CT surgery based on developing innovative events and whether events could change a student's perception of the specialty based on event type and summation of event attendance.

Previous research assessing the impact of a CT surgery interest group at a large academic institution noted an increase in interest and better understanding of the field after events were conducted. Additionally, the study focused on developing preset events for students that included a relaxed environment to interact with CT surgery faculty, simulation wet laboratory sessions, lecture events, and shadowing opportunities [7]. This study was successful in increasing CT surgery interest and provided a framework for other large academic institutions. However, a recent study by the Thoracic Surgery Medical Student Association determined that most medical students are not connected to a large allopathic academic institution and assessed the needs of medical students across the nation who are interested in CT surgery. The study concluded that there is a significant need for medical student engagement due to limited exposure at home institutions which then defined the purpose of the national association [12].

Our data, in accordance with Davis and colleagues, suggests that there are certain types of events that are more enjoyable for students and help them understand the field of CT surgery to a greater extent [7]. Conducting wet lab events such as vascular anastomosis or heart wet labs was highly rated as the most enjoyable events and increased a student's confidence in their ability to perform new skills. These same events, in addition to the lecture given by a DO surgical resident, were events that students would like to see repeated in subsequent years. Certain events such as lectures by CT, cardiac, and congenital cardiac surgeons, the heart wet lab, and discussion with the MS4 were events where students seemed to learn something new about CT surgery. Furthermore, having the discussion with the MS4 and congenital cardiac surgeon was rated the highest for helping students feel more prepared to apply for CT surgery residency. Our group believes that this may be due to the MS4's relatability.

Events focusing on recruiting alumni from a school's program or a clinical medical student who has had success in the specialty of interest may be an event that increases interest the most. This may be due to the connection that pre-clinical students may have with these trainees who are currently or have gone through the same path as them. Furthermore, the DO general surgical resident may have increased interest because osteopathic medical students can resonate with the resident's struggles and path to pursue a field in surgery. By giving students the opportunity to learn from those ahead of them, they can gain a further understanding of the field.

While there has been an increase in osteopathic students applying to surgical subspecialties, their match rate in all surgical subspecialties remains significantly lower than their allopathic counterparts [13]. This has led to a significantly smaller ratio of osteopathic physicians in surgery and, consequently, a lower number of osteopathic surgeons involved in academia [14]. Interventions to minimize these discrepancies may involve students seeking opportunities in research or showing early interest and dedication in a specific surgical subspecialty. Our study attempts to complete the latter by giving students an introduction and multiple opportunities to be exposed to CT surgery.

Our previous study, in accordance with Coyan and colleagues, determined the negative factors associated with CT surgery at our institution [4,5]. This novel part of this study attempted to determine if event attendance could change perceptions of CT surgery and quantify whether greater change in perception correlated with more events attended. Focusing on the negative factors previously determined to be associated with CT surgery, our data exhibited that the local cardiac surgeon school preceptor consistently developed a positive perception when analyzing personalities of CT surgeons, work/life balance, and a CT surgeon having time for their family. Similar to data from Davis and colleagues, events that can bring in a CT surgeon to speak in-person and in a relaxed environment may be the strongest way to change perceptions of the field [7]. However, this study was unique in that it used a privately employed cardiac surgeon instead of someone affiliated with academia. While the committee had a congenital cardiac surgeon who is associated with an academic program speak, it may be more influential for schools that are not connected to an academic program to recruit surgeons who work in private practice to further simulate how a student may see themselves in the future. Furthermore, the Thoracic Surgery Directors Association stated that most job offers in CT surgery for residents were in private practice [15]. What may be most interesting about this study is the ability to ameliorate the negative perceptions of CT surgery as a student attends more events hosted by the committee. By gaining full exposure to the field by hearing from surgeons at different levels in their career and those that relate to a student's path to getting simulation experience through wet labs, students' negative perceptions of work/life balance and lack of family time were altered significantly.

Limitations

This study has important limitations to consider. Aside from the fact that it was conducted at a single osteopathic medical school that focuses on producing primary care physicians, this study was also conducted over one academic year and was not a longitudinal study following students from pre-clinical to clinical years. With the size of the sample, we recognize that there may be a response bias. Furthermore, one event that the committee hoped to conduct was to invite a CT surgery resident of the allopathic or osteopathic profession. Based on our data, we believe that this event would have increased interest and helped students feel even more prepared to apply to a CT surgery residency.

## Conclusions

While negative perceptions associated with CT surgery exist, these may be ameliorated with more exposure to the field. Unique events that expose pre-clinical students to multiple facets of CT surgery as well as offering hands-on activities may increase interest in the field and may lead to further pursuit of the field during clinical years. Furthermore, future research should be dedicated to following students over their medical school journey to determine if interest developed during pre-clinical years correlates to them pursuing CT surgery. Finally, we hope this study provides a model for medical schools that lack the resources of a large academic institution to develop a CT surgery interest group that exposes students to the field, increases interest across their pre-clinical years, and ultimately defies the negative perceptions associated with CT surgery through innovative and student-tailored events.

## Appendices

**Table 1:** Survey questions CT: cardiothoracic; OMS: osteopathic medical student

Survey questions	
1. Class year	a. OMS-I
	b. OMS-II
2. Gender	a. Male
	b. Female
	c. Other
	d. Prefer not to answer
3. Race/ethnicity	a. Caucasian
	b. African-American
	c. Latino or Hispanic
	d. Asian
	e. Native American
	f. Native Hawaiian or Pacific Islander
	g. Two or more
	h. Other/unknown
	i. Prefer not to say
4. What is your age? (free response)	"Typed response"
5. Did you start medical school immediately after college?	a. Yes
	b. No
6. Did you have a previous career before attending medical school?	a. Yes
	b. No
7. What is your relationship status?	a. Single
	b. Married/domestic partnership
	c. Divorced/widowed
	d. Prefer not to say
8. Type in the title of the event you are attending (free response)	"Typed response"
9. How many CT surgery events have you attended?	a. 1
	b. 2
	c. 3
	d. 4
	e. 5
	f. 6
	g. 7
10. Please rate the following statements using the scale below. (Likert 5-point scale) (Each option will include: 1. Strongly disagree, 2. Somewhat disagree 3. Neither agree nor disagree, 4. Somewhat agree, 5. Strongly agree)	a. CT surgeons have a good work/life balance
	b. CT surgeons have personalities that are easy to get along with
	c. CT surgery is a growing field and will continue to thrive
	d. CT surgeons are leaders of the healthcare team
	e. CT surgeons are appropriately compensated for the work they perform
	f. CT surgeons have time for their families
11. Please complete the following statements based on your experience from this event. (Likert 5-point scale) (Each option will include: 1. Strongly disagree, 2. Somewhat disagree 3. Neither agree nor disagree, 4. Somewhat agree, 5. Strongly agree)	a. This event increased my interest in CT surgery
	b. I have a better understanding of CT surgery because of this event
	c. I would like to see more events like this in the future
	d. I learned something new about CT surgery from this event
	e. This event increased my confidence in my ability to perform new skills
	f. This event was enjoyable
	g. I feel more prepared to apply to a CT surgery residency program if I chose to
